# On the Deleterious and Medicinal Effects of Green Tea

**Published:** 1818-06

**Authors:** 


### *On the deleterious and medicinal Effects of Green Tea.* By EDWARD PERCIVAL, M. D.

[Dublin Hospital Reports, vol. i.]

THE intrinsic properties of TEA, as a universal beverage, are little ascertained; its deleterious effects are overlooked, or regarded with indifference; and its medicinal virtues are not dreamt of by the physician. Dr. Percival's attention was drawn towards the subject by a case, illustrating the mode in which green tea, when taken in excess, may operate as a poison, which occurred about twelve years ago, the particulars of which we shall briefly sketch.

CASE. A gentleman travelling in Devonshire in a hot summer's day, drank green tea in considerable quantities three or four times, eating little else than some biscuit. Soon after retiring to bed, he began to feel some unusual and distressing sensations about the præcordia, as though he were about to faint. In this way he passed two hours in a kind of troubled slumber. His respiration now became irregular and oppressed, with palpitation of the heart, and irregularity of its action. He started up suddenly, as if from Incubus, with acute spasmodic pain in the region of the heart, and a constant sense of deliquium. The pulse was feeble and intermittent, and slight fits of apparent asphyxia occurred every five or six minutes. He procured from a fellow traveller two grains of opium, and a little cold brandy and water, from which he derived some relief, and he again composed himself to sleep. In an hour he awoke in the same state of agitation as before, gasping for breath and bedewed with a chilly moisture. Another grain of opium was procured, and a glass of warm brandy and water swallowed. These brought relief; he fell into a sound sleep, and awoke in perfect health. Though unaccustomed to opium, the three grains he had taken left no head-ache, or other symptom usually following opium.

CASE 2. A somewhat similar case occurred to Dr. Harvey, upwards of thirty years ago. In the middle of the day, a medical gentleman rapped at the door, requesting permission to come in and die at the Doctor's house. He appeared in great agitation and alarm; his pulse was extremely irregular, and scarcely perceptible; he was confident he was dying. He acknowledged that he had sat up the whole of the preceding night, and drank much strong green tea. Dr. H. immediately gave him a glass of cherry brandy, and put him to bed. He slept two hours, and awoke in good health.

From these data it follows, that "green tea possesses a specific power of controlling and *abating* the motions of the heart." 223. Dr. Darwin recommended cold tea as a grateful and salutary drink in febrile diseases, and Dr. Percival confirms the justice of Dr. D's advice. The restlessness and hurried circulation, he observes, which attend suppurative fevers, especially pulmonary phthisis, are greatly alleviated by green tea, especially if given in a diluted state. Its power over the action of the heart and arteries, Dr. P. thinks nearly equal to that of digitalis; while in allaying nervous irritability it may be compared with opium, cicuta, or hyoscyamus. It is free from the nauseating qualities of the digitalis, and the constipating effects of the opium. It is also diuretic, and therefore in hydropic cases green tea is a useful auxiliary. If the spirit of juniper be added to the infusion, its diuretic powers are much increased.

It is somewhat curious, yet it is equally certain, that tea, and especially green tea, restores regularity to a pulse which is habitually the reverse. This our author has observed in many instances, both in health and in disease. There is no objection to its use, therefore, in hydrothorax, or organic disease of the heart, though some of our great physicians make a point of prohibiting its use in such cases.

The efficacy of opium and brandy in counteracting the mischief occasioned by the excessive use of green tea, suggests the expedient of employing that infusion as a remedy for an over dose of opium. Coffee affords relief where the dose has not been immoderate, but green tea is more powerful.

Without attempting to invalidate any of Dr. Percival's positions relative to the *good* qualities of tea, we think he has not dwelt sufficiently on the peculiarly *antisoporific* effects of this delicious beverage. There are very few people of much susceptibility of nerve who can procure sleep after an evening dose of green tea. For our own parts, it has so invariably brought on *Incubus*, that we have long been obliged to give up its use. It is, however, a cordial of the most refreshing nature, and we believe it to be possessed of *all* the virtues enumerated by Dr. Percival, and many of those so celebrated in the epic poem of the Chinese monarch; yet we have reason to know, that these virtues are not without their alloy, and that nervous or highly sensitive patients will do well to watch the effects of green tea on the functions of their nervous systems before they indulge freely in so luxurious a beverage.

---

